# miRNome Profiling Detects miR-101-3p and miR-142-5p as Putative Blood Biomarkers of Frailty Syndrome

**DOI:** 10.3390/genes13020231

**Published:** 2022-01-26

**Authors:** Giulia Carini, Jessica Mingardi, Francesco Bolzetta, Alberto Cester, Andrea Bolner, Giampietro Nordera, Luca La Via, Alessandro Ieraci, Isabella Russo, Stefania Maggi, Stefano Calza, Maurizio Popoli, Nicola Veronese, Laura Musazzi, Alessandro Barbon

**Affiliations:** 1 Department of Molecular and Translational Medicine, University of Brescia, 25123 Brescia, Italy; giulia.carini@unibs.it (G.C.); j.mingardi001@unibs.it (J.M.); luca.lavia@unibs.it (L.L.V.); isabella.russo@unibs.it (I.R.); stefano.calza@unibs.it (S.C.); 2 Genetics Unit, IRCCS Istituto Centro S. Giovanni di Dio Fatebenefratelli, 25125 Brescia, Italy; 3 Medical Department, Geriatric Unit, Azienda ULSS (Unità Locale Socio Sanitaria), 3 Serenissima, 30174 Venice, Italy; francesco.bolzetta@gmail.com (F.B.); alberto.cester55@gmail.com (A.C.); 4 CSOX-Centro Stress Ossidativo, 36057 Arcugnano, Italy; bolner.andrea@gmail.com (A.B.); giampi.nordera@gmail.com (G.N.); 5 Department of Pharmaceutical Sciences, University of Milan, 20133 Milan, Italy; alessandro.ieraci@unimi.it (A.I.); maurizio.popoli@unimi.it (M.P.); 6 Aging Branch, Neuroscience Institute, National Research Council, 35100 Padua, Italy; stefania.maggi@in.cnr.it (S.M.); ilmannato@gmail.com (N.V.); 7 Geriatrics Section, Department of Medicine, University of Palermo, 90121 Palermo, Italy; 8 School of Medicine and Surgery, University of Milano-Bicocca, 20126 Milan, Italy; laura.musazzi@unimib.it

**Keywords:** microRNA, frailty, smRNA-seq, miRNome, biomarkers, RNA-seq, miR-101-3p, miR-142-5p

## Abstract

Frailty is an aging-related pathology, defined as a state of increased vulnerability to stressors, leading to a limited capacity to meet homeostatic demands. Extracellular microRNAs (miRNAs) were proposed as potential biomarkers of various disease conditions, including age-related pathologies. The primary objective of this study was to identify blood miRNAs that could serve as potential biomarkers and candidate mechanisms of frailty. Using the Fried index, we enrolled 22 robust and 19 frail subjects. Blood and urine samples were analysed for several biochemical parameters. We observed that sTNF-R was robustly upregulated in the frail group, indicating the presence of an inflammatory state. Further, by RNA-seq, we profiled 2654 mature miRNAs in the whole blood of the two groups. Expression levels of selected differentially expressed miRNAs were validated by qPCR, and target prediction analyses were performed for the dysregulated miRNAs. We identified 2 miRNAs able to significantly differentiate frail patients from robust subjects. Both miR-101-3p and miR-142-5p were found to be downregulated in the frail vs. robust group. Finally, using bioinformatics targets prediction tools, we explored the potential molecular mechanisms and cellular pathways regulated by the two miRNAs and potentially involved in frailty.

## 1. Introduction

Frailty is an aging-related condition, generally defined as a state of increased vulnerability to endogenous and exogenous stressors that results from a decreased physiological reserve in multiple organs and systems [1,2]. Frail subjects have a limited capacity to meet homeostatic demands and a high risk of developing adverse health outcomes [3]. The prevalence of frailty has been assessed in many studies worldwide using a variety of frailty measures. Although the results are highly variable, the overall prevalence has been estimated at around 11–16% in the population 60 years and older [4,5]. Frailty is more prevalent in women compared to men, and the prevalence increases with age, being the highest in subjects over 85 years [4,6]. The majority of studies are based on the definition of frailty introduced by Fried and collaborators in 2001, which considers frailty as a clinical syndrome in which three or more of the following criteria are present: unintentional weight loss, fatigue or self-reported exhaustion, weakness, slow walking speed, and reduced or absent physical activity [2,7]. 

In the last years, many efforts have been made to understand the molecular mechanisms underlying frailty [8] and to find biomarkers for a correct diagnosis [9]. Research evidence suggests that, among the cellular mechanisms that might underlie frailty, senescence, oxidative stress, mitochondrial dysfunctions, and inflammation have major roles in frailty pathophysiology [10,11]. However, despite the progress in identifying biomarkers of frailty in recent years, currently there is not a clear consensus. Different studies have identified promising candidates including markers across the immune system, endocrine system, clinical blood markers, proteins, markers of oxidative damage, and epigenetic markers [8,12,13]. Among the latter, microRNAs (miRNAs) are emerging as promising non-invasive diagnostic and prognostic biomarkers, as well as potential therapeutic agents [14]. Importantly, miRNAs have been proposed as both peripheral biomarkers and potential molecular factors involved in physiological and pathological aging [15,16,17]. Thanks to the miRNA ability to target hundreds of transcripts at once, mainly repressing translation or inducing mRNA degradation of target transcripts through sequence-specific binding [18], miRNAs are key fine-tuning regulators in most physiological processes [19,20]. Thus, it should not be surprising that miRNAs are recognized as key modulators of virtually all physiological processes and, consequently, miRNA dysregulation has been reported in a multiplicity of clinical conditions [14]. To date, only two studies have investigated changes in miRNA expressions in frail subjects [21,22]. These two studies identified several miRNAs as possible novel candidate biomarkers for frailty in old age, without overlap in their results [23]. 

Research into the epigenetics of frailty could be very useful, not only for the identification of potential frailty biomarkers but also to understand the underlying mechanisms of frailty and aging. Indeed, miRNAs are emerging as promising potential therapeutic agents, given their role as novel regulators of the human protein-coding genes [24].

In the present work, we analyzed the whole miRNome of frail vs. robust subjects and identified miR-101-3p and miR-142-5p as specifically down-regulated in frail patients. Furthermore, using bioinformatic target prediction tools, we explored the potential molecular mechanisms and cellular pathways regulated by these two miRNAs that might be potentially involved in frailty.

## 2. Materials and Methods

### 2.1. Patients’ Recruitment and Clinical Assessment

This study was approved by the local Ethical Committee, registration number “91 A/CESC 16/10/2018”, and performed following the Declaration of Helsinki principles. All the subjects were recruited at ULSS 3 “Dolo” (Venezia) and clinically evaluated by expert clinicians in geriatric medicine. As inclusion criteria, we considered both genders and age > 70 years, while the exclusion criteria were life expectancy of fewer than 12 months or the presence of an acute or chronic condition that could interfere with the study outcomes (e.g., heart failure in NYHA class 3–4, severe renal or liver failure, dementia, major depression, or other relevant neurological/psychiatric diseases). After obtaining the participant’s informed consent, a questionnaire regarding past and recent medical history and medications was administered. Then, a physical examination was performed, including measurements of weight and height. 

Patient classification in the robust and frail was based on Fried’s criteria [7]: (I) unintentional weight loss > 5% in the last year; (II) weakness, as measured by handgrip strength; (III) slow gait speed over 4 m of walking; (IV) exhaustion, ascertained by asking the question on the 30-item Geriatric Depression Scale (GDS), “Do you feel full of energy?”, considering participants as exhausted if they gave a negative answer and also had a GDS score ≥ 10; and (V) low energy expenditure, defined as weekly physical activity below 383 kcal/week in males and 270 kcal/week in females, as calculated through the PASE (Physical Activity Scale for the Elderly) [25]. People having at least three of these criteria were defined as frail [7]. A Mini-Mental State Examination (MMSE) was applied to all subjects to evaluate their cognitive status, and only subjects with a score ≥ 20/30 were included in the study [26]. Frail and robust subjects were age- and sex-matched. Other clinical tests were performed to characterize the patient cohorts by assessing autonomy in basic activities of daily living (ADL) and instrumental activities of daily living (Instrumental-ADL), quality of life (Short Form 12), and comorbidities (Cumulative Illness Rating Scale, CIRS).

### 2.2. Biochemical Analyses

Blood and urine samples were obtained from subjects to analyze biochemical parameters. Serum/plasma protein levels (total protein (PRO), albumin (ALB)) were measured to evaluate general health; creatinine (CRE), urea, uric acid (UA), glomerular filtration rate test (GFR), alkaline phosphatase (ALP), alanine aminotransferase (ALT), aspartate aminotransferase (AST), and γ-glutamyl transferase (GGT) were measured to evaluate kidney and liver functions; high-sensitivity C-reactive protein (hsCRP), Interleukin-1 (IL-1), Interleukin-6 (IL-6), tumor necrosis factor α (TNFalpha), and soluble tumor necrosis factor receptor (sTNF-R) were measured to evaluate inflammatory state; plasma 3-nitrotyrosine (3NT), total glutathione (GSH tot), reduced glutathione (GSH rid), GSH ratio (GSH rid/GSH tot), biological antioxidant potential (BAP), REDOX index (normalized ROM/BAP ratio), serum reactive oxygen metabolites (ROM), 8-hydroxy-deoxyguanosine (U-8OHdG), 2-deoxyguanosine (U-2dG), and ratio U-8OHdG/U-2dG were measured to evaluate oxidative stress level. All the parameters were measured by HPLC, colorimetric or fluorometric methods, according to standard procedures. Statistical analyses were carried out using GraphPad Prism version 8.2.1 program (GraphPad software, Inc, San Diego, CA, USA); the statistical significance between groups was determined using two-tailed *t*-test, and the differences were considered significant with a *p* value < 0.05 (*), <0.01 (**), <0.001 (***) and <0.0001 (****).

### 2.3. miRNome Sequencing and Analysis

Whole blood of robust and frail subjects was collected in the PAXgene Blood RNA Tube (PreAnalytiX GmbH, Hombrechtikon, Switzerland), and total RNA enriched of small RNAs (smRNAs) was extracted using the PAXgene Blood miRNA kit (PreAnalytiX GmbH) according to the manufacturer’s protocol. RNA quality control was assessed on an Agilent 2100 BioAnalyzer (Agilent Technologies, Santa Clara, CA, USA). Small RNA sequencing (smRNA-seq) was performed using the Illumina technology platform. smRNA-seq analysis was performed starting from 250 ng of total RNA per sample using the SMARTer smRNA-Seq kit (Takara Bio USA, Inc, San Jose, CA, USA) according to the manufacturer’s protocol. Briefly, the protocol starts with a first phase of polyadenylation in which a poly(A) tail is added to the starting RNA. Subsequently, reverse transcription is carried out using an oligo (dT) primer which allows the incorporation of an adaptive sequence at the 5′ end of each single-stranded cDNA molecule. Furthermore, when the retro-transcriptase enzyme reaches the 3′ end of each RNA template, it adds some non-complementary nucleotides which are recognized by a second primer, the SMART smRNA Oligo, which allows the addition of a second adaptive sequence at the 3′ end of each single-stranded cDNA molecule. cDNA is thus amplified using specific primers that recognize the adapter sequences inserted during the retro-transcription phase which allow for the insertion of two additional adapters. The PCR products are subsequently purified, and the obtained libraries are quantified and qualitatively evaluated by capillary electrophoresis to verify that the fragment size is correct for the subsequent sequencing step. The amplified products are sequenced on the Illumina platform, based on Solexa technology (Illumina, Inc., San Diego, CA, USA). The sequencing takes place through chemical synthesis with terminators which are added automatically, and when a cluster incorporates one, there will be an emission of fluorescence detected by the instrument.

Subsequently, bioinformatic analysis was performed on the raw data obtained from sequencing. After the alignment of the raw data to the reference database (miRbase Release 22.1: October 2018, which contains 38,589 miRNAs, of which 1917 miRNA precursors and 2654 mature miRNAs for Homo sapiens (GRCh38) [27]) the stably expressed miRNAs were selected by applying a cut-off of at least 10 reads in 50% of patients in each group. To detect differentially expressed miRNAs between the frail and robust groups, a negative binomial regression considering several biological covariates (i.e., BMI, smoking, alcohol, pharmacological therapy) was used as a criterion for the selection of miRNAs. To assess significant differences in the miRNome expression profile in frail subjects, we selected miRNAs with log_2_(FC) > |1| a *p* value < 0.05. Multiple testing correction was applied to control the false-discovery rate (FDR) using the Benjamini–Hochberg (BH) procedure. miRNAs with an FDR < 0.25 were selected and retained for further analysis. 

### 2.4. Validation of smRNA-Seq Expression Data by Quantitative PCR and Statistical Analyses

Blood level expression of selected miRNAs was evaluated by quantitative PCR (qPCR) in each sample, using the TaqMan Advanced miRNA Assay kit (Thermo Fisher Scientific, Waltham, MA, USA) according to the manufacturer’s protocol on ABI PRISM 7500 sequence detection system (Thermo Fisher Scientific, Waltham, MA, USA). To analyze the qPCR data, the averages of the CT values of each miRNA measured in triplicate in robust and frail subjects, determined by the 7500 system SDS software (version 1.3.1, Life Technologies) were used. The data were expressed as the variation of the log2 fold in the frail subjects compared to the robust samples. Based on RNA-seq data, miR-486-5p, identified as the most stable expressed miRNA, was used as an endogenous control to normalize each sample. The expression values obtained were used for the statistical analysis carried out with GraphPad Prism, version 8.2.1 (GraphPad Software, www.graphpad.com, accessed on 23 December 2021); the statistical significance between groups was determined using two-tailed *t*-tests, and the differences were considered significant with a *p* value < 0.05 (*), <0.01 (**), <0.001 (***) and <0.0001 (****). The receiving operating characteristic (ROC) curve was used to assess the ability of differentially expressed miRNAs to distinguish frail from robust subjects.

### 2.5. Bioinformatic Targets Prediction

Potential mRNA targets of differentially expressed miRNAs were determined by bioinformatic analysis. The miRNAs’ targets were determined by integrating the results of three software: TargetScan (http://www.targetscan.org, accessed on 27 August 2021) [28], MicroT-CDS (http://www.microrna.gr/microT-CDS/, accessed on 27 August 2021) [29], and MirDB (http://mirdb.org, accessed on 27 August 2021) [30]; the miRNA targets predicted by at least two of the three software packages were retained for further bioinformatic analyses. 

The EnrichR web server (http://amp.pharm.mssm.edu/Enrichr, accessed on 6 October 2021) [31,32] was queried for the identification of significantly enriched functional annotations, Gene Ontology (GO), biological processes and molecular functions, and pathway analysis using the integrated databases KEGG [33] and PANTHER [34]. Specifically, the main GO categories and pathways were examined with an FDR < 20%.

In addition, the target genes were compared with the genes present in the Aging Atlas (https://ngdc.cncb.ac.cn/aging/index, accessed on 31 August 2021) [35] that reports data on genes/proteins involved in aging biology.

## 3. Results

### 3.1. Patient Evaluation and Biochemical Analyses

A total of 41 subjects over 70 years of age and matched for gender, were recruited at ULSS 3 “Dolo” (Venezia) by expert clinicians in geriatric medicine, following the Fried’s index and the MMSE to assess the cognitive domain (only subjects with MMSE over 20/30 were included in the study). People having at least three of Fried’s criteria were defined as frail, while the others were placed in the robust group. By applying these criteria, 22 subjects were classified as robust, and 19 as frail (Table S1). As expected, the frail subjects performed worst in the tests to assess the autonomy in basic ADL (*p* = 0.01) and IADL (*p* < 0.0001), and had less PCS (*p* < 0.0001) while having the same MSC as robust subjects (Table S2). Moreover, as reported in Table S2, several biochemical parameters were measured in the patients’ serum/plasma and urine samples. Among all the biochemical parameters analyzed, only the plasma level of soluble tumor necrosis factor receptor was found to be dysregulated (1.7-fold increase in frail patients *p* = 0.01). Trends of variations were observed also for albumin (*p* = 0.05), total protein level (*p* = 0.05), glomerular filtration rate (*p* = 0.06), and 3-nitrotyrosine (*p* = 0.09).

### 3.2. Human Blood miRNome Profiling Identifies 9 miRNAs Differentially Expressed in Frail Compared to Robust Subjects

To find selectively up-or down-regulated miRNAs associated with frailty, we compared the whole miRNome of frail subjects to that of the robust group to find selectively up-or down-regulated miRNAs in whole blood. We tested 2654 mature miRNAs. By selecting small RNAs with at least 10 reads in 50% of the patients in each group, we identified 210 mature miRNAs. Applying a log2FC > |1|, a *p* < 0.05, and an FDR of 25%, 9 miRNAs were found to be differentially expressed between the frail and robust groups. Among these, 7 miRNAs were down-regulated while two were up-regulated (Table 1).

### 3.3. Validation of RNA-Seq Expression Data by Quantitative PCR

**Figure 1:**
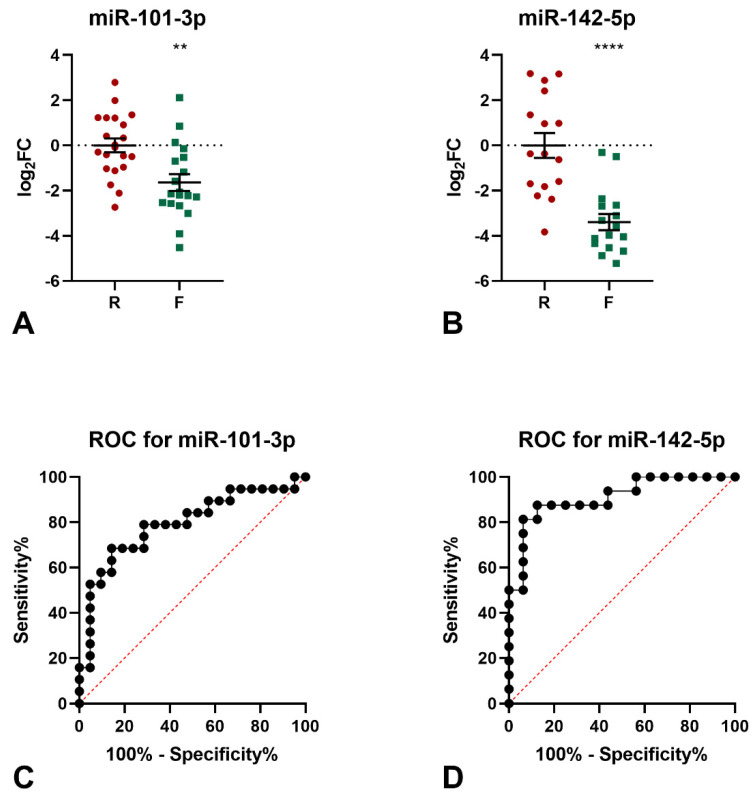
Results of qPCR analyses of miR-101-3p and mir-142-5p. (**A**) qPCR analysis of miR-101-3p expression. Two-tailed *t*-test: ** *p* < 0.01 F vs. R. (**B**) qPCR analysis of miR-142-5p expression. Two-tailed *t*-test: **** *p* < 0.0001 F vs. R. ROC analysis for the ability of miR-101-3p levels (**C**) and miR-142-5p levels (**D**) to discriminate between frail and robust subjects.

The levels of the 9 miRNAs identified by miRNomic analysis were measured by qPCR (not shown) and the differential expression in the frail samples of miR-101-3p and miR-142-5p was confirmed (Figure 1A,B). Indeed, both miRNAs were found to be down-regulated in the frail group, as compared to robust subjects (miR-101-3p: logFC −1.64, *p* = 0.0014; miR-142-5p: logFC −3.40, *p* < 0.0001). The diagnostic significance of miR-101-3p was further tested by ROC curve analysis. The calculated area under the ROC curve (AUC) of 0.792 (95% CI = 0.6463−0.9376; *p* = 0.016) indicated that the levels of this miRNA may discriminate frail from robust subjects with good accuracy in the examined cohort (Figure 1C). Similarly, the calculated AUC of 0.882 (95% CI = 0.7664−0.9983; *p* = 0.0002) for miR-142-5p indicated that the levels of this miRNA may discriminate frail from robust subjects with excellent accuracy in the examined cohort (Figure 1D).

### 3.4. Bioinformatic Analysis of miR-101-3p Target Genes

**Figure 2:**
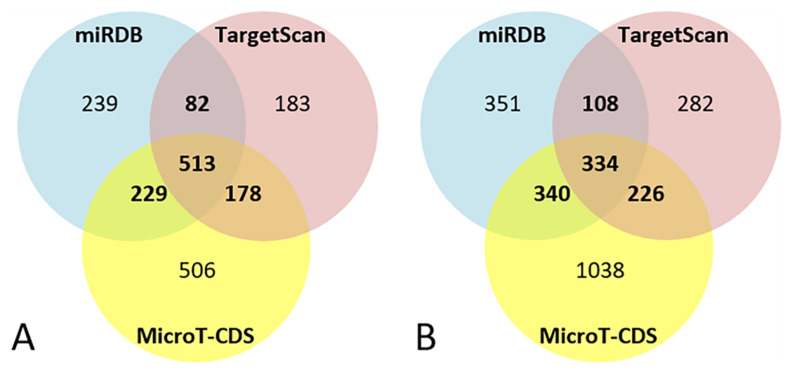
Venn diagrams of bioinformatics target prediction for miR-101-3p (**A**) and miR-142-5p (**B**).

For miR-101-3p, the bioinformatics target prediction analysis identified 1063 targets from miRDB, 956 from TargetScan, and 1428 from MicroT-CDS. Of these, 1002 target genes were predicted by at least two of the three software packages, and 513 were predicted by all of them (Figure 2A).

As reported in Table 2, the analysis of the pathways of the 1002 targets of miR-101-3p highlighted the involvement of numerous genes in significant neuronal processes such as axon guidance and the formation and regulation of dopaminergic and cholinergic synapses. Various signal transduction pathways were also involved, including PI3K-Akt pathway, Erb and MTOR signaling, and the insulin/IGF pathway, in addition to the signal cascade mediated by TGF-β, Wnt, EGF, FGF, MAPK, Ras, and cAMP. Moreover, several genes were implicated in the regulation of cellular senescence, apoptosis, and signaling pathways regulating pluripotency of stem cells. Finally, various genes were involved in the inflammatory response. Regarding the GO analysis, many genes were involved in basic cellular mechanisms such as the regulation of transcription and gene expression, protein modification, intracellular signaling, and cell migration (Table 2). 

### 3.5. Bioinformatic Analysis of miR-142-5p Target Genes

For miR-142-5p the bioinformatics target prediction analysis identified 1135 targets from miRDB, 950 from TargetScan, and 1939 from MicroT-CDS. Of these, 1008 target genes were predicted by at least two of the three software packages, and 334 were predicted by all of them (Figure 2B).

Pathway analysis of the 1008 targets of miR-142-5p highlighted that several genes were involved in brain functions, such as axon guidance, formation and regulation of dopaminergic and cholinergic synapses, glutamatergic synapse, and metabotropic glutamate receptor activity. Various signal transduction pathways were also involved, including TGF-β signaling, signaling mediated by MAPK, the insulin/IGF pathway, and Erb and mTOR signaling. Moreover, inflammation mediated by chemokine and cytokine signaling pathways was identified as associated with miR-142-5p targets. The GO analysis shows that most genes were involved in the maintenance of cellular mechanisms such as regulation of transcription and gene expression, cell migration and cytoskeleton organization, protein modification, and intracellular signaling transduction (Table 3). 

### 3.6. Bioinformatic Analysis of miRNAs’ Target Genes Related to Aging

**Figure 3:**
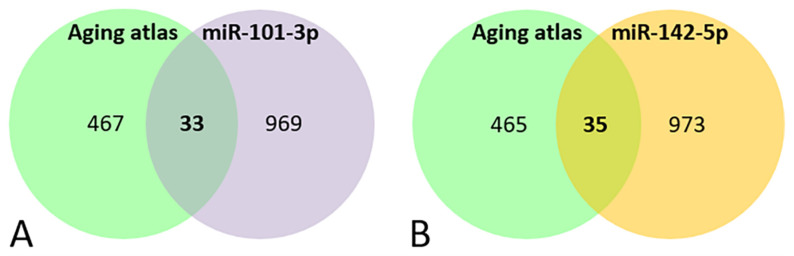
Venn diagram of the genes present in the aging databases and the target predicted for miR-101-3p (**A**) and miR-142-5p (**B**).

A Venn diagram shows that 33 genes out of the 1002 miR-101-3p targets, and 35 out of the 1008 targets of miR-142-5p were involved in aging processes (Figure 3A, Table 4A and Figure 3B, Table 4B respectively).

**Figure 4:**
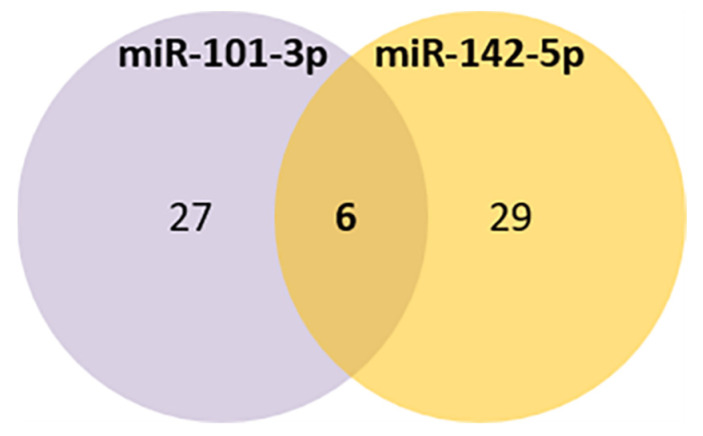
Venn diagram of the common aging targets predicted for miR-101-3p and miR-142-5p.

Comparing the selected miRNAs target genes and the aging database, 6 aging-related genes were found to be potentially targeted by both miRNAs (*LRP2*, *GSK3B*, *NOG*, *RORA*, *CREB1*, *EIF5A2*) (Figure 4, Table 4C).

## 4. Discussion

In the present study, we profiled clinical and biochemical parameters and sequenced the entire miRNome from 41 old subjects divided into two cohorts: 22 robust and 19 frail individuals. The goal was the identification of possible biomarkers, pathways, and molecular mechanisms underlying the pathophysiology of frailty. Among the biochemical parameters analyzed, we observed that sTNF-R, the soluble receptor of the pro-inflammatory cytokine TNFalpha, was robustly upregulated in the frail group. Furthermore, whole blood miRNome analysis, followed by validation with qPCR, identified miR-101-3p and miR-142-5p as strongly downregulated in frail subjects. These two miRNAs could be considered potential biomarkers of frailty.

Frailty is a common condition in the old people, defined as a state of increased vulnerability to stressors that can cause a limited ability to recover [7]. Clinical features of frailty are associated with age-related chronic inflammation (inflammaging), oxidative stress, mitochondrial dysfunctions, insulin resistance, aging-related loss of anabolic hormones, diminished strength, and diminished tolerance to physical activity [10,11]. Nevertheless, the molecular mechanisms underlying frailty are still largely unknown.

Previous studies have analyzed several biochemical parameters as potential frailty biomarkers [36,37,38]. However, no clear common deregulated parameters have been identified to date [8]. Specifically, in our study, among all the biochemical parameters analyzed, only the plasma levels of sTNF-R were significantly different in frail subjects compared to robust subjects. The upregulation of sTNF-R1 levels was previously associated with frailty [39,40]. TNFR1 belongs to the TNF-receptor superfamily; it is constitutively expressed on most cell types and is activated by TNFalpha. The intracellular signaling mediated by TNFR1 can mediate both cell survival and apoptosis through the activation of the NF-κB, JNK, and p38 pathways or Caspase 8, respectively. Moreover, it functions as a regulator of inflammation [41,42]. The strong upregulation of sTNF-R reported in the frail group might indicate the presence of an inflammatory state, a typical pathological condition present in frailty. 

Recently, the study of miRNAs as putative biomarkers of aging diseases has gained interest. miRNAs regulate several biological events related to the aging process, but their expression is also influenced by aging processes themselves. At the same time, miRNAs have been consistently linked to the main systemic and cellular processes associated with frailty, including inflammaging [16,43,44,45], cellular senescence [46,47,48], skeletal muscle maintenance and energetic metabolisms [15,49,50], and the maintenance of brain and neuronal functions [51,52,53,54]. 

To the best of our knowledge, only two studies to date have investigated changes in miRNA expressions in frail subjects [22,55]. In the first such work, Ipson and collaborators evaluated the changes to the plasma-derived exosome miRNA profiles of young, old robust, and old frail individuals, finding eight miRNAs specifically dysregulated in the frail group (miR-10a-3p, miR-92a-3p, miR-185-3p, miR-194-5p, miR-326, miR-532-5p, miR-576-5p, and miR-760) [22]. The second study evaluated the levels of three inflammation-related miRNAs and one miRNA related to the control of melatonin synthesis in the plasma of healthy adults, older robust, and older frail subjects. Among these, miR-21 had a higher expression level in frail subjects than controls [55]. Although very preliminary, these two studies identified a few miRNAs as possible novel candidate biomarkers for frailty. Furthermore, other miRNAs related to inflammaging, musculoskeletal system and muscle wasting, and mitochondrial miRNAs have been proposed as candidates for an early diagnosis of frailty [21]. 

Our miRNome profiling by whole blood RNA-seq analysis allowed for the identification of 9 miRNAs differentially expressed in frail subjects compared to robust ones. The observed differences in the RNA-seq of two miRNAs were confirmed by qPCR: miR-101-3p and miR-142-5p, which appear to be robustly down-regulated in frail subjects, compared to robust subjects. Interestingly, in previous studies, the downregulation in the expression levels of both of these two miRNAs was related to aging [16,56,57].

Of the two validated miRNAs, miR-101-3p has been extensively studied in cancer [58]. It is known to regulate oxidative stress-induced apoptosis of breast cancer cells [59], proliferation and apoptosis in osteosarcoma cells [60], and autophagy and apoptosis in hepatocellular carcinoma cells [61]. Moreover, miR-101-3p was reported to play a key role in regulating cell senescence [62,63], inflammation [64], and aging-related disorders such as diabetes [65,66] and Parkinson disease [67]. 

Concerning miR-142-5p, its expression has been associated with immune and inflammatory responses and diseases [68,69,70,71], as well as with muscle maintenance and homeostasis [72,73]. Moreover, it was found to play a role in the maintenance of redox homeostasis [74,75]. In cancer, miR-142-5p suppresses proliferation and promotes apoptosis of the human osteosarcoma cell line [76], promotes proliferation, invasion, and migration in breast cancer [77], and suppresses tumorigenesis in non-small cell lung cancer [78]. 

The functional classification of the target genes of the two miRNAs has shown their involvement in processes such as neural cell development and maintenance, and regulation of stem cell pluripotency and cell senescence in addition to several intracellular signaling pathways whose dysregulation could be linked to the pathophysiology of frailty. 

It should be noted that sTNF-R, which we found to be up-regulated in the frail group, is not a predicted target of either miR-101-3p or miR-142-5p. Thus, other molecular pathways may be involved in sTNF-R level regulation.

Among the target genes also reported in the aging database with a potential role in aging processes, six were targeted by both miRNAs and are aging-related genes (*LRP2*, *GSK3B*, *NOG*, *RORA*, *CREB1*, *EIF5A2*). Of these, *LRP2* encoding for the low-density lipoprotein receptor-related protein 2 or megalin, is an endocytic receptor expressed on the apical surface of epithelial cells that internalizes a variety of ligands, including nutrients, hormones, signaling molecules, and extracellular matrix proteins [79]. Due to the important roles of its ligands and its wide tissue expression pattern, megalin has been recognized as an important component of many pathological conditions [79,80]. *GSK3B* encodes for the Glycogen Synthase Kinase 3 β, a key regulator of several cellular functions, including growth signaling, cell fate, cell senescence, inflammation, and metabolism [81]. Due to its major role in several cellular processes, changes in *GSK3B* expression and function are strongly related to several aging-related diseases such as diabetes, cancer, inflammatory conditions, and neurodegenerative disorders [81,82]. *NOG*, or Noggin, is a binding partner of BMPs (bone morphogenetic proteins) and an antagonist of BMP signaling. Noggin is involved in the development of many body tissues, including nerve tissue, muscles, and bones [83]. Moreover, it was shown to have a major role in cell senescence. Indeed, the injection of Noggin into SAMP8 mice, a senescence-associated strain used to model aspects of aging, restored neurogenesis and neural stem cell counts [84]. CREB1, CAMP responsive element binding protein 1, is a protein that binds the cAMP response element and regulates transcription. Its expression and function can modulate oxidative stress-induced senescence in granulosa cells by reducing the mitochondrial function [85] and CREB signaling has been related to lung and brain aging [86,87]. Finally, *EIF5A2* encoding for the Eukaryotic Initiation Factor 5A2, plays a key role in the regulation of protein translation, and it has been reported that, in transgenic mice, its overexpression enhances the aging process [88].

## 5. Conclusions

Overall, the results obtained here represent one of the first approaches for studying the involvement of miRNAs in the pathophysiology of frailty. Moreover, we observed that sTNF-R was robustly upregulated in the frail group, suggesting the presence of an inflammatory state, a typical pathological condition present in frailty. 

However, we recognize that this work might have some limitations. First, our findings are derived from a limited number of subjects, implying that they should be confirmed in a larger cohort of patients. Second, smRNA-seq was carried out on whole blood samples, while plasma and serum have differences in the expression levels of some miRNAs, and it would be interesting to evaluate the expression of miR-101-3p and 142-5p in these blood components specifically. Finally, the miRNA target genes discussed here are only bioinformatically predicted; their real interactions should be confirmed in the appropriate biological samples. 

More studies are warranted to confirm the validity of miR-101-3p and 142-5p as peripheral biomarkers and/or molecular effectors of frailty. The effective validation of the roles of certain miRNAs, including miR-101-3p and miR-142-5p, and their potential target mRNAs could shed light on the biological and molecular mechanisms underlying this condition and assist in the development of both preventive and treatment interventions exploiting these specific miRNAs as possible biomarkers.

## Figures and Tables

**Table 1:** miRNAs identified as differentially expressed by RNA-seq analysis in frail vs. robust patients.

miRNA	logFC	*p* Value	FDR
hsa-miR-101-3p	−2.23	2.71 × 10^−9^	1.39 × 10^−6^
hsa-miR-16-2-3p	−1.71	4.67 × 10^−9^	1.39 × 10^−6^
hsa-miR-19a-3p	−1.69	3.17 × 10^−8^	6.28 × 10^−6^
hsa-miR-144-5p	−1.67	2.92 × 10^−7^	4.33 × 10^−5^
hsa-miR-126-5p	−1.21	2.20 × 10^−4^	2.09 × 10^−2^
hsa-miR-142-5p	−1.10	7.92 × 10^−5^	9.41 × 10^−3^
hsa-miR-19b-3p	−1.01	2.47 × 10^−4^	2.09 × 10^−2^
hsa-miR-125b-5p	1.09	8.28 × 10^−4^	5.47 × 10^−2^
hsa-miR-5690	1.20	4.23 × 10^−4^	3.14 × 10^−2^

**Table 2:** Bioinformatic analysis of miR-101-3p target genes. The table reports the 20 most representative terms of pathways and GO analysis of miR-101-3p target genes.

TOP 20 KEGG Pathways			
Term	Overlap	*p* Value	FDR
Axon guidance	26/182	1.40 × 10^−6^	3.74 × 10^−4^
PI3K-Akt signaling pathway	35/354	1.04 × 10^−4^	0.006
Focal adhesion	23/201	1.93 × 10^−4^	0.007
MAPK signaling pathway	29/294	4.08 × 10^−4^	0.014
Ras signaling pathway	24/232	0.001	0.019
TGF-β signaling pathway	13/94	0.001	0.020
Parathyroid hormone synthesis, secretion and action	14/106	0.001	0.020
Dopaminergic synapse	16/132	0.001	0.021
GnRH secretion	10/64	0.001	0.021
ErbB signaling pathway	12/85	0.001	0.021
cAMP signaling pathway	22/216	0.001	0.021
Sphingolipid signaling pathway	14/119	0.003	0.038
Inositol phosphate metabolism	10/73	0.003	0.041
Phosphatidylinositol signaling system	12/97	0.003	0.041
Thyroid hormone signaling pathway	14/121	0.003	0.041
AGE-RAGE signaling pathway in diabetic complications	12/100	0.004	0.044
Cholinergic synapse	13/113	0.004	0.044
Endocytosis	23/252	0.004	0.044
Signaling pathways regulating pluripotency of stem cells	15/143	0.005	0.050
Cellular senescence	16/156	0.005	0.050
**TOP 20 PANTHER Pathways**			
**Term**	**Overlap**	***p* Value**	**FDR**
EGF receptor signaling pathway Homo sapiens P00018	16/109	1.04 × 10^−4^	0.006
Wnt signaling pathway Homo sapiens P00057	29/278	1.59 × 10^−4^	0.006
FGF signaling pathway Homo sapiens P00021	14/99	4.09 × 10^−4^	0.007
Integrin signaling pathway Homo sapiens P00034	19/156	3.07 × 10^−4^	0.007
CCKR signaling map ST Homo sapiens P06959	19/165	0.001	0.009
PI3 kinase pathway Homo sapiens P00048	7/42	0.005	0.046
VEGF signaling pathway Homo sapiens P00056	8/54	0.005	0.047
Alzheimer disease-amyloid secretase pathway Homo sapiens P00003	8/56	0.006	0.052
Ras Pathway Homo sapiens P04393	9/69	0.007	0.053
Alzheimer disease-presenilin pathway Homo sapiens P00004	11/99	0.011	0.064
PDGF signaling pathway Homo sapiens P00047	12/112	0.010	0.064
Oxytocin receptor mediated signaling pathway Homo sapiens P04391	6/39	0.012	0.064
Endothelin signaling pathway Homo sapiens P00019	9/75	0.012	0.064
Apoptosis signaling pathway Homo sapiens P00006	10/102	0.032	0.113
Hypoxia response via HIF activation Homo sapiens P00030	4/24	0.030	0.113
Insulin/IGF pathway-protein kinase B signaling cascade Homo sapiens P00033	5/34	0.026	0.113
5HT2 type receptor mediated signaling pathway Homo sapiens P04374	6/46	0.026	0.113
T-cell activation Homo sapiens P00053	8/73	0.029	0.113
Interleukin signaling pathway Homo sapiens P00036	9/86	0.028	0.113
Cadherin signaling pathway Homo sapiens P00012	13/150	0.038	0.122
**TOP 20 GO Biological Process**			
**Term**	**Overlap**	***p* Value**	**FDR**
regulation of transcription by RNA polymerase II (GO:0006357)	209/2206	1.27 × 10^−20^	4.68 × 10^−17^
regulation of transcription, DNA-templated (GO:0006355)	197/2244	8.24 × 10^−16^	7.61 × 10^−13^
regulation of gene expression (GO:0010468)	103/1079	1.67 × 10^−10^	6.87 × 10^−8^
protein phosphorylation (GO:0006468)	58/496	1.69 × 10^−9^	4.82 × 10^−7^
cellular protein modification process (GO:0006464)	96/1025	1.99 × 10^−9^	5.26 × 10^−7^
epithelial to mesenchymal transition (GO:0001837)	13/47	3.33 × 10^−7^	7.23 × 10^−5^
negative regulation of transmembrane receptor protein serine/threonine kinase signaling pathway (GO:0090101)	20/108	3.63 × 10^−7^	7.45 × 10^−5^
mesenchymal cell differentiation (GO:0048762)	13/51	9.34 × 10^−7^	1.73 × 10^−4^
blood vessel morphogenesis (GO:0048514)	13/56	2.94 × 10^−6^	4.93 × 10^−4^
chromatin remodeling (GO:0006338)	18/103	3.30 × 10^−6^	0.001
regulation of BMP signaling pathway (GO:0030510)	15/76	4.55 × 10^−6^	0.001
regulation of transforming growth factor β receptor signaling pathway (GO:0017015)	17/100	9.00 × 10^−6^	0.001
negative regulation of cell migration (GO:0030336)	21/144	1.02 × 10^−5^	0.001
regulation of cellular macromolecule biosynthetic process (GO:2000112)	46/468	1.05 × 10^−5^	0.001
regulation of microtubule polymerization (GO:0031113)	10/40	2.04 × 10^−5^	0.003
protein localization to nucleus (GO:0034504)	17/106	1.99 × 10^−5^	0.003
neuron migration (GO:0001764)	11/50	2.92 × 10^−5^	0.003
chromatin organization (GO:0006325)	20/142	2.76 × 10^−5^	0.003
generation of neurons (GO:0048699)	25/202	2.84 × 10^−5^	0.003
axonogenesis (GO:0007409)	28/240	2.89 × 10^−5^	0.003
**TOP 20 GO Molecular Function**			
**Term**	**Overlap**	***p* Value**	**FDR**
protein serine/threonine kinase activity (GO:0004674)	47/344	4.00 × 10^−10^	2.70 × 10^−7^
sequence-specific double-stranded DNA binding (GO:1990837)	75/712	8.63 × 10^−10^	2.88 × 10^−7^
RNA polymerase II cis-regulatory region sequence-specific DNA binding (GO:0000978)	105/1149	1.28 × 10^−9^	2.88 × 10^−7^
cis-regulatory region sequence-specific DNA binding (GO:0000987)	104/1149	2.61 × 10^−9^	4.39 × 10^−7^
RNA polymerase II transcription regulatory region sequence-specific DNA binding (GO:0000977)	117/1359	4.57 × 10^−9^	6.15 × 10^−7^
sequence-specific DNA binding (GO:0043565)	67/707	4.01 × 10^−7^	4.50 × 10^−5^
transcription regulatory region nucleic acid binding (GO:0001067)	29/212	8.81 × 10^−7^	8.47 × 10^−5^
nuclear import signal receptor activity (GO:0061608)	7/16	5.96 × 10^−6^	4.71 × 10^−4^
DNA binding (GO:0003677)	70/811	6.30 × 10^−6^	4.71 × 10^−4^
transcription cis-regulatory region binding (GO:0000976)	52/549	8.18 × 10^−6^	0.001
DNA-binding transcription activator activity, RNA polymerase II-specific (GO:0001228)	36/333	1.25 × 10^−5^	0.001
double-stranded DNA binding (GO:0003690)	58/651	1.58 × 10^−5^	0.001
nuclear localization sequence binding (GO:0008139)	7/24	1.27 × 10^−4^	0.007
mRNA binding (GO:0003729)	28/263	1.45 × 10^−4^	0.007
nuclear receptor binding (GO:0016922)	16/120	3.23 × 10^−4^	0.015
DNA-binding transcription repressor activity, RNA polymerase II-specific (GO:0001227)	26/256	0.001	0.022
GTPase regulator activity (GO:0030695)	24/233	0.001	0.027
adenyl ribonucleotide binding (GO:0032559)	29/306	0.001	0.029
histone demethylase activity (H3-K27 specific) (GO:0071558)	3/5	0.001	0.041
zinc ion binding (GO:0008270)	30/336	0.002	0.054

**Table 3:** Bioinformatic analysis of miR-142-5p target genes. The table reports the most representative terms of pathways and the GO analysis of miR-142-5p target genes.

TOP 20 KEGG Pathways			
Term	Overlap	*p* Value	FDR
Axon guidance	29/182	3.57 × 10^−8^	9.47 × 10^−6^
Signaling pathways regulating pluripotency of stem cells	20/143	3.33 × 10^−5^	0.003
Endocytosis	29/252	3.02 × 10^−5^	0.003
Ubiquitin mediated proteolysis	19/140	7.88 × 10^−5^	0.005
Regulation of actin cytoskeleton	25/218	1.10 × 10^−4^	0.006
TGF-β signaling pathway	14/94	2.51 × 10^−4^	0.007
MAPK signaling pathway	30/294	2.02 × 10^−4^	0.007
Dopaminergic synapse	16/132	0.001	0.022
Cholinergic synapse	14/113	0.002	0.031
Oxytocin signaling pathway	17/154	0.002	0.035
Aldosterone-regulated sodium reabsorption	7/37	0.002	0.037
ErbB signaling pathway	11/85	0.004	0.046
Phosphatidylinositol signaling system	12/97	0.003	0.046
Thyroid hormone signaling pathway	14/121	0.003	0.046
Hippo signaling pathway	17/163	0.004	0.046
Glutamatergic synapse	13/114	0.005	0.056
mTOR signaling pathway	16/154	0.005	0.056
Long-term potentiation	9/67	0.006	0.066
Hedgehog signaling pathway	8/56	0.007	0.068
Sphingolipid signaling pathway	13/119	0.007	0.069
**PANTHER Pathways**			
**Term**	**Overlap**	***p* Value**	**FDR**
PDGF signaling pathway Homo sapiens P00047	15/112	4.98 × 10^−4^	0.040
Ras Pathway Homo sapiens P04393	10/69	0.002	0.075
Alzheimer disease-presenilin pathway Homo sapiens P00004	12/99	0.004	0.075
CCKR signaling map ST Homo sapiens P06959	17/165	0.004	0.075
Angiogenesis Homo sapiens P00005	15/142	0.005	0.075
Hypoxia response via HIF activation Homo sapiens P00030	5/24	0.006	0.075
Insulin/IGF pathway-protein kinase B signaling cascade Homo sapiens P00033	6/34	0.006	0.075
Vasopressin synthesis Homo sapiens P04395	3/10	0.012	0.117
TGF-β signaling pathway Homo sapiens P00052	10/88	0.013	0.117
Inflammation mediated by chemokine and cytokine signaling pathway Homo sapiens P00031	17/188	0.014	0.117
Metabotropic glutamate receptor group III pathway Homo sapiens P00039	7/54	0.018	0.134
Ubiquitin proteasome pathway Homo sapiens P00060	6/43	0.020	0.135
**TOP 20 GO Biological Process**			
**Term**	**Overlap**	***p* Value**	**FDR**
regulation of transcription by RNA polymerase II (GO:0006357)	183/2206	3.58 × 10^−12^	8.72 × 10^−9^
regulation of transcription, DNA-templated (GO:0006355)	185/2244	4.57 × 10^−12^	8.72 × 10^−9^
positive regulation of cell differentiation (GO:0045597)	33/258	8.64 × 10^−7^	0.001
regulation of cell migration (GO:0030334)	43/408	4.15 × 10^−6^	0.002
ubiquitin-dependent protein catabolic process (GO:0006511)	38/354	9.75 × 10^−6^	0.005
protein phosphorylation (GO:0006468)	48/496	1.20 × 10^−5^	0.005
actin cytoskeleton reorganization (GO:0031532)	13/63	1.25 × 10^−5^	0.005
phosphorylation (GO:0016310)	41/400	1.35 × 10^−5^	0.005
proteasome-mediated ubiquitin-dependent protein catabolic process (GO:0043161)	35/321	1.55 × 10^−5^	0.005
sensory organ development (GO:0007423)	12/56	1.80 × 10^−5^	0.005
regulation of BMP signaling pathway (GO:0030510)	14/76	2.30 × 10^−5^	0.006
visual system development (GO:0150063)	10/41	2.71 × 10^−5^	0.007
modification-dependent protein catabolic process (GO:0019941)	25/201	2.88 × 10^−5^	0.007
peptidyl-threonine phosphorylation (GO:0018107)	12/60	3.74 × 10^−5^	0.008
negative regulation of cellular response to growth factor stimulus (GO:0090288)	14/80	4.18 × 10^−5^	0.008
regulation of cytoskeleton organization (GO:0051493)	17/112	4.46 × 10^−5^	0.008
protein ubiquitination (GO:0016567)	48/525	5.15 × 10^−5^	0.009
positive regulation of RIG-I signaling pathway (GO:1900246)	5/10	6.56 × 10^−5^	0.011
nervous system development (GO:0007399)	42/447	8.14 × 10^−5^	0.012
mRNA destabilization (GO:0061157)	9/38	8.78 × 10^−5^	0.012
**TOP 20 GO Molecular Function**			
**Term**	**Overlap**	***p* Value**	**FDR**
ubiquitin-protein transferase activity (GO:0004842)	46/392	8.92 × 10^−8^	6.15 × 10^−5^
sequence-specific double-stranded DNA binding (GO:1990837)	66/712	1.31 × 10^−6^	4.52 × 10^−4^
ubiquitin protein ligase activity (GO:0061630)	32/263	3.73 × 10^−6^	0.001
protein serine/threonine kinase activity (GO:0004674)	35/344	6.57 × 10^−5^	0.006
mRNA 3′-UTR binding (GO:0003730)	14/85	8.34 × 10^−5^	0.007
myosin binding (GO:0017022)	11/56	9.31 × 10^−5^	0.007
nuclear receptor binding (GO:0016922)	17/120	1.08 × 10^−4^	0.007
purine ribonucleoside triphosphate binding (GO:0035639)	42/460	1.53 × 10^−4^	0.010
DNA-binding transcription activator activity, RNA polymerase II-specific (GO:0001228)	33/333	1.73 × 10^−4^	0.010
transcription cis-regulatory region binding (GO:0000976)	47/549	2.87 × 10^−4^	0.015
RNA polymerase II transcription regulatory region sequence-specific DNA binding (GO:0000977)	97/1359	3.04 × 10^−4^	0.015
RNA polymerase II cis-regulatory region sequence-specific DNA binding (GO:0000978)	84/1149	3.70 × 10^−4^	0.017
GDP binding (GO:0019003)	11/67	4.85 × 10^−4^	0.020
kinase activity (GO:0016301)	15/112	4.98 × 10^−4^	0.020
GTPase binding (GO:0051020)	22/201	0.001	0.020
cis-regulatory region sequence-specific DNA binding (GO:0000987)	83/1149	0.001	0.021
mRNA 3’-UTR AU-rich region binding (GO:0035925)	6/22	0.001	0.021
myosin V binding (GO:0031489)	5/15	0.001	0.021
guanylate kinase activity (GO:0004385)	4/9	0.001	0.021
mRNA binding (GO:0003729)	26/263	0.001	0.024

**Table 4:** The table reports the miR-101-3p and miR-142-5p target genes that are in common with the aging atlas database. (**A**) Common miRNAs between miR-101-3p targets and those present in the aging atlas database. (**B**) Common miRNAs between miR-142-5p targets and those present in the aging atlas database. (**C**) Common miRNAs between miR-101-3p and miR-142-5p target genes and those present in the aging atlas database.

**A**	mir-101-3p targets present in aging atlas	*PLCG1*, *ADH5*, *GCLM*, *KL*, *PIK3CB*, *FGFR3*, *PRKAA1*, *MTOR*, *ELN*, *JAK2*, *PTGS2*, *EIF5A2*, *ITGA2*, *GSK3B*, *FOXO1*, *GCLC*, *SESN3*, *LMNB1*, *RORA*, *TNFSF11*, *CREB1*, *FOS*, *CXCL12*, *LRP2*, *CEBPA*, *SOCS2*, *NOG*, *CXCL6*, *MXD1*, *AKT3*, *HGF*, *APP*, *TOP1*
**B**	miR-142-5p targets present in aging atlas	*PRKCB*, *NRG1*, *IGF1*, *RPS6KA5*, *RICTOR*, *HSPA8*, *NFE2L2*, *BMP2*, *GSK3B*, *TNFAIP3*, *SIRT7*, *RB1CC1*, *CREB1*, *LRP2*, *PDGFRA*, *NBN*, *PTEN*, *ULK1*, *KRAS*, *PAPPA*, *SUN1*, *EIF5A2*, *TOPORS*, *CLOCK*, *PRKAA2*, *RORA*, *FGF7*, *MXI1*, *PIK3CA*, *PSAT1*, *TNFSF13B*, *IL6ST*, *NOG*, *APPL1*, *PRKCA*
**C**	miR-101-3p and miR-142-5p targets present in aging atlas	*LRP2*, *GSK3B*, *NOG*, *RORA*, *CREB1*, *EIF5A2*

## Data Availability

All data presented in this study are not publicly available following the ethical guidelines. The data presented are available upon request from the corresponding author.

## Supplementary Materials

The following supporting information can be downloaded at: https://www.mdpi.com/article/10.3390/genes13020231/s1, Table S1: Clinical features of the recruited subjects; Table S2: Statistical analyses of clinical and biochemical data of the subjects included in the study.
