# Supplementary material for: miRNome Profiling Detects miR-101-3p and miR-142-5p as Putative Blood Biomarkers of Frailty Syndrome
Source: Genes (Basel). 2022 Jan 26;13(2):231. doi: 10.3390/genes13020231 (PMC8872439; doi:10.3390/genes13020231)
Supplement: Supplementary file 1 [file genes-13-00231-s001.zip › genes-1546679-supplementary.pdf]

Table S1. Clinical features of the recruited subjects.

Subject	Group	Sex	Age (years)	Weight (Kg)	Height (cm)	BMI (kg/m2)	Handgrip (Kg)	Walking speed (s)	Weight loss (Kg)	PASE	GDS	MMSE	ADL	IADL	PCS	MCS
1	1	1	75	74	165	27.4	16.9	3.6	2	226.0	0	21.0	6	8	47.8	51.4
3	1	1	72	62	155	25.8	24.4	5.1	7	242.0	12	22.3	6	6	49.6	44.6
4	1	2	82	85	175	27.7	27.6	3.1	0	231.3	2	27.7	6	5	53.5	44.9
12	2	2	91	80	165	29.6	11.2	19.0	2	40.0	0	24.0	4	2	20.9	46.0
13	1	1	90	45	150	20.0	17.6	5.0	0	114.5	7	28.0	6	7	43.6	49.2
14	2	1	82	55	158	22.0	5.8	na	0	62.9	11	24.0	4	0	31.4	42.2
17	1	2	76	86	168	30.4	22.7	2.9	0	217.0	0	26.7	6	5	56.1	45.4
18	2	1	86	51	155	21.2	11.8	5.7	6	0.0	0	25.2	6	4	30.8	51.3
19	1	1	85	56	155	23.3	13.2	5.0	0	187.0	0	25.4	6	8	30.7	48.1
21	1	1	82	80	165	29.4	18.6	na	10	216.0	4	26.4	6	8	43.3	57.5
22	2	1	78	66	155	27.5	15.3	8.7	5	111.0	11	25.0	6	8	36.4	25.7
23	1	2	77	76	175	25.3	34.5	2.0	0	263.0	0	27.0	6	5	55.9	55.9
24	1	2	77	78	175	26.0	21.4	3.3	0	106.0	0	28.7	6	4	43.3	57.5
25	1	1	74	72	160	28.1	16.9	4.4	0	111.0	4	27.3	5	6	41.6	57.6
26	1	1	84	65	160	23.0	14.9	5.0	0	131.0	0	29.4	6	8	45.7	44.2
27	2	1	81	77	155	32.0	7.2	9.3	8	136.7	11	25.4	6	8	22.9	51.0
28	2	1	87	92	150	40.0	14.4	11.7	0	60.0	0	25.2	5	1	20.6	49.8
30	2	1	84	68	168	26.5	15.1	9.8	0	40.0	0	22.4	4	0	31.2	40.1
33	1	1	76	60	155	25.0	16.4	3.8	0	194.5	1	28.0	6	8	46.6	52.8
34	1	2	83	65	160	25.4	27.3	2.2	0	201.0	0	26.1	6	5	55.3	60.7
35	2	1	85	60	158	24.0	7.5	na	6	20.0	0	22.4	4	0	28.3	48.9
36	1	1	80	50	144	24.1	16.2	7.9	0	151.0	12	28.4	6	8	51.9	28.3
37	1	2	86	67	165	24.8	20.4	3.3	3	523.0	7	30.0	6	4	40.4	55.2
39	2	1	83	73	158	30.0	7.7	13.0	0	40.0	12	25.7	5	7	26.9	35.2
41	2	1	82	74	160	28.9	10.2	11.5	5	60.0	0	26.4	5	3	29.4	48.3
42	1	1	82	78	160	30.4	18.3	3.2	0	187.0	4	29.5	6	6	51.0	43.1
43	2	1	92	65	160	25.4	12.7	8.1	0	40.0	0	23.2	6	6	27.7	48.1
45	1	2	76	75	175	24.5	34.9	2.7	0	161.0	13	25.7	6	5	51.5	34.5
46	1	1	79	76	160	29.7	15.8	3.5	0	148.1	0	26.7	6	8	40.6	54.4

47	2	1	78	76	150	33.7	12.6	3.6	0	66.7	13	28.7	6	3	49.0	48.9
48	2	2	79	54	168	19.3	28.0	5.1	10	69.5	0	23.0	6	0	43.9	37.3
49	2	2	85	90	177	28.7	21.1	9.0	10	40.0	0	23.4	6	3	35.2	47.8
50	2	2	81	65	160	25.4	29.4	4.8	7	54.3	0	26.4	6	5	35.6	52.9
52	1	2	90	76	170	26.3	29.6	4.2	0	42.9	0	26.0	6	2	44.5	53.7
53	1	1	90	50	150	22.2	11.1	8.5	0	126.0	0	23.2	5	5	44.9	58.2
55	1	1	83	52	160	20.3	18.1	na	0	146.0	0	22.4	6	7	54.9	44.8
56	2	2	87	65	150	29.3	17.2	8.5	2	40.0	0	23.2	6	1	27.6	47.8
58	1	1	86	62	156	25.8	19.0	5.4	0	111.0	0	23.4	5	5	44.3	57.0
60	2	2	77	78	172	26.9	15.1	7.8	0	20.0	8	23.3	5	3	26.7	40.1
61	2	2	77	58	170	20.0	27.5	6.1	6	40.0	0	28.7	6	5	49.3	51.4
62	2	2	81	64	160	25.0	11.6	6.0	0	34.3	7	24.4	6	4	26.9	34.6

Group: 1=Robust, 2=Frail. Sex: 1=Female, 2=Male. Body Mass Index (BMI), Physical Activity Scale for the Elderly (PASE), Geriatric Depression Scale (GDS), Mini-Mental State Examination (MMSE), Activities of Daily Living (ADL), Instrumental Activity of Daily Living (IADL), Physical Component Summary (PCS), Mental Component Summary (MCS), not assessed (na).

Table S2. Statistical analyses of clinical and biochemical data of the subjects included in the study.

Parameter	Units	Robust (n=20)	Frail (n=19)	p-value
**Age**	years	82 (4.9)	82.9 (4.4)	na
**Female sex**	-	12/20	11/19	na
**BMI**	kg/m2	25.6 (3.0)	27.1 (5.0)	na
**ADL**	-	5.9 (0.3)	5.4 (0.8)	0.01
**IADL**	-	6.1 (1.8)	3.3 (2.7)	**<0.0001**
**PCS**	-	47.3 (6.5)	31.6 (8.3)	**<0.0001**
**MCS**	-	49.8 (8.3)	44.6 (7.3)	0.07
**UA**	mg/dL	5.2 (1.7)	5.3 (1.7)	0.88
**U-UA**	mg/dL	26.2 (9.9)	22.4 (12.4)	0.31
**ALB**	g/L	4.3 (0.3)	4.0 (0.5)	0.05
**ALT**	U/L	11.1 (3.4)	12.9 (8.2)	0.39
**AST**	U/L	20.4 (4.0)	19.6 (5.1)	0.61
**CRE**	mg/dL	0.9 (0.4)	1.0 (0.3)	0.40
**U-CRE**	g/L	0.9 (0.4)	0.8 (0.6)	0.80
**ALP**	U/L	80.3 (19.8)	87.6 (32.1)	0.40
**GGT**	U/L	28.1 (29.2)	43.9 (42.4)	0.18
**hsCRP**	mg/L	3.0 (3.5)	6.9 (10.6)	0.15
**PRO**	g/L	7.0 (0.4)	6.7 (0.5)	0.05
**GFR**	mL/min	84.3 (27.9)	68.1 (21.8)	0.06
**UREA**	mg/dL	43.4 (15.6)	53.0 (19.8)	0.10
**GSHtot**	umol/L	1254.2 (262.5)	1169.7 (309.5)	0.37
**GSHrid**	umol/L	1104.5 (234.7)	10728 (257.4)	0.69
**GSHratio**	%	88.5 (8.3)	92.5 (7.6)	0.13
**BAP**	uEq/L	2361.4 (273.9)	2387.2 (251.8)	0.77
**ROM**	Ucarr	371.4 (42.6)	363.5 (47.1)	0.59
**REDOX index**	norm. ratio	0.9 (0.1)	0.9 (0.2)	0.46
**3NT**	ug/L	231.9 (600.3)	623.4 (791.5)	0.09
**U-8OHdG**	ug/g creat	167.2 (499.9)	3941.3 (12510.4)	0.22
**U-8OHdG_U**	ug/g urate	343.5 (868.0)	14007.1 (44926.1)	0.19
**U-2dG**	ug/g creat	227.7 (462.1)	241.3 (529.9)	0.93
**U-2dG_U**	ug/g urate	1132.9 (2822.1)	1759.6 (5574.5)	0.67
**U8OH2dG/2dG**	ng/ug	191.5 (815.2)	5616.9 (21450.5)	0.28
**TNFalpha**	ng/L	28.0 (37.4)	20.0 (14.5)	0.40
**sTNF-R**	ug/L	3.6 (1.7)	6.1 (3.4)	**0.01**
**IL-1**	ng/L	0.6 (0.8)	0.9 (1.1)	0.44
**IL-6**	ng/L	3.1 (2.4)	3.5 (2.5)	0.59

Biochemical samples from two robust patients were not available. Data are reported as mean (S.D.). Two-tailed t-test; significant p-values are reported in bold. Body Mass Index (BMI), Activities of Daily Living (ADL), Instrumental Activity of Daily Living (IADL), Physical Component Summary (PCS), Mental Component Summary (MCS), uric acid (UA), urinary uric acid (U-UA), albumin (ALB), alanine aminotransferase (ALT), aspartate aminotransferase (AST), creatinine (CRE), urinary creatinine (U-CRE), alkaline phosphatase (ALP), gamma glutamyl transferase (GGT), high-sensitivity C-reactive protein (hsCRP), total protein (PRO), glomerular filtration rate test (GFR), total glutathione (GSH tot), reduced glutathione (GSH rid), GSH ratio (GSH rid/GSH tot), biological antioxidant potential (BAP), reactive oxygen metabolites (ROM), plasma 3-nitrotyrosine (3NT), urinary 8-hydroxy-deoxyguanosine (U-8OHdG), urinary 2-deoxyguanosine (U-2dG), tumor necrosis factor alpha (TNFalpha), soluble tumor necrosis factor receptor (sTNF-R), Interleukin-1 (IL-1), Interleukin-6 (IL-6).
